# 

**Published:** 1896-10

**Authors:** 


### CONTENTS.

ORIGINAL ARTICLES—

Surgical Immunization Compared with Susceptibility and Predisposition to Infection. By J. McFadden Gaston, M. D., Atlanta, Ga.......... 481

Puerperal Eclampsia as a Result of Albuminuria in the Parturient State. By A. A. Smith, M. D., Hawkinsville, Ga...... 495

Resection of Gasserian Ganglion and Extirpation of Trigeminus. By Dr. Paul Poirier, Paris.......... 498

Some of the Unsuspected Effects of the Bromides. By J. Allison Hodges, M. D., Richmond, Va.......... 505

ORIGINAL ARTICLES—(Continued.)

Adeps Lanae—An Ideal Ointment Base. By Bernard Wolff, M. D., Atlanta, Ga. ..... 513

SOCIETY REPORTS—

Richmond Academy of Medicine and Surgery ..... 516

EDITORIAL— 519

SPECIAL NOTES— 526

PRESCRIPTIONS— 533

